# A Study Comparing the Efficacy of “The All-on-Four Implants” in the Maxillary and the Mandibular Arches

**DOI:** 10.7759/cureus.87197

**Published:** 2025-07-02

**Authors:** Daisy Loyola, Anuradha Navaneetham, S Surraj, Satish Kumaran P

**Affiliations:** 1 Oral and Maxillofacial Surgery, Etihad Airways Medical Center, Abu Dhabi, ARE; 2 Oral and Maxillofacial Surgery, Hospital for Orthopaedics, Sports Medicine, Arthroplasty, and Trauma (HOSMAT) Hospital, Bengaluru, IND; 3 Anatomy, All India Institute of Medical Sciences (AIIMS) Hyderabad, Hyderabad, IND; 4 Oral and Maxillofacial Surgery, Mathrusri Ramabai Ambedkar Dental College & Hospital, Bengaluru, IND

**Keywords:** abutments, all on 4, implants, marginal bone loss, stability

## Abstract

Introduction

The “all-on-four” technique of dental implants has been in popular use for the past few years due to its ease of rehabilitation of teeth. This technique employed four simultaneous implants over the upper maxillary and lower mandibular arches to suit the needs of the patient as a prosthesis. However, the comparative utility of this technique of implants between the maxillary and the mandibular arches has not been thoroughly evaluated. Hence, this study bridged that gap and compared the impact of this technique in the upper and lower jaw arches.

Aim and objectives

The main aim of this study was to comparatively assess the impact and compatibility of the “all-on-four” technique in both the maxillary and the mandibular arches after the implant procedure. The objectives of this study were to compare and assess the mean pocket depths as well as the mean crestal bone losses in both the maxillary and mandibular arches after the implant procedure. Also, this study compared and assessed the implant stability quotients as well as the gingival indices in both the maxillary and mandibular arches after the implant procedure.

Methodology

This was a prospective study done on 90 patients who were followed up to 24 months after the procedure, with intervals ranging from 3 months, 6 months, 12 months, 18 months, and 24 months after the completion of the procedure. Muco-periosteal flaps were raised from one molar to the contralateral molar, accompanied by vertical releasing incisions after a thorough assessment using orthopantomographs and cone beams. This was then followed by impressions and placement of guiding wires using abutment devices. Then the implants were aligned to guide them and position them at four quadrants in each arch. During the follow-up of this procedure, the averages of the pocket depths (mm), crestal bone loss (mm), and implant stability quotients, as well as the gingival indices, were compared between the upper and lower arches after the placement of implants.

Results

The values of mean crestal bone loss (mm) and mean pocket depths (mm) were higher in maxillary implants compared to the mandibular implants, and the aforementioned values became stabilized in mandibular implants from 12 to 24 months of follow-up, compared to the maxillary implants. The gingival indices were healthier in mandibular implants compared to their maxillary counterparts. The implant stability quotient values were higher in mandibular implants compared to the maxillary implants.

Conclusions

In this study, the mandibular implants were found to be better adapted to the “all-on-four” procedural technique. The stability quotients of the mandibular implants were also much better compared to their maxillary counterparts, thereby suggesting the possibility that the lower jaw arch is a more promising site for this technique than the upper jaw arch.

## Introduction

Dental implants have not only served as prosthetic replacements for the missing teeth of edentulous patients but have also improved the oral functions in them, apart from preserving the tensile strength of their teeth [1,2]. These implants had not only helped patients with hollowed teeth to recover and preserve their teeth for longer periods of time but had also helped in maintaining their structure and shape in addition to maintaining the fitness of their jawbones [2]. Though there were different techniques available that could help the oral surgeons insert the dental implants in the right place, the ‘all-on-four technique’ of dental implants stood out as it had gained popularity over the past few years due to its aesthetic appeal, reduced healing time, and stability [2,3]. Being a fixed prosthetic replacement procedure, this ‘all-on-four technique’ of dental implants utilized non-removable dentures conditioned with peripherally fitted abutment plates that served to replace the entire arch of either the maxilla or the mandible with four implants at a time, which helped to restore the oral functions and confidence in patients by preserving their gingivae and preventing significant tooth loss in them [3]. However, the efficacy of this technique of ‘all-on-four implants’ between the maxilla and the mandible had not been validated yet, as there were not many comparative studies done to substantiate the same [2,4]. The superiority of these implants in the upper jaw as compared to those of the lower jaw, or vice versa, was also yet to be established, validated, and compared [3,4]. Hence, this study served to address the aforementioned problem and helped to bridge that gap with substantial evidence that proved the significant utility of these implants in the mandibular arches and that the “all-on-four-implants” technique was more effective in the lower jaw arch than in the upper jaw, thereby making it a potentially suitable prosthesis for the lower jaw as compared to the upper jaw. The main aim of this study was to comparatively assess the impact and compatibility of the “all-on-four” technique in both the maxillary and mandibular arches after the implant procedure. The objectives of this study were to compare and assess the mean pocket depths as well as the mean crestal bone losses in both the maxillary and mandibular arches after the implant procedure. Also, this study compared and assessed the implant stability quotients as well as the gingival indices in both the maxillary and mandibular arches after the implant procedure.

## Materials and methods

This was a prospective study that was done for a period of six years, between 2018 and 2024, on 90 patients. This study included the follow-up of patients at 3 months, 6 months, 12 months, and 18 months, respectively, from the time of completion of the procedure. Each patient was rehabilitated with four implants in the upper arch and another four in the lower arch. All the procedures that were performed in this study were conducted only after obtaining approval from the Ethics Committee of the place where this study was conducted. The sample size of 90 patients was estimated by the free “clinical sample size estimation software” available online from the website [15]. A population size of 102 was fed into the software based on the reference sample size of a previous related study [4]. After feeding this value into the software for a confidence interval of 95% with a 5% margin of error, the expected proportion of the population was kept at 50, and the software had deduced the sample size to be 80 patients. Keeping an attrition factor of 10, the sample size arrived at 90 patients in total. A hand-written, proper, informed patient consent was taken prior to the start of surgery, after explaining all the complications of the procedure to the patients in their local language. This study included only those patients who needed complete rehabilitation of both their maxilla and their mandibles, either due to edentulous teeth, poor bone mineralization, or tooth loss. This study included only those patients with acceptable oral hygiene, measured using the standard debris index score ranging from 0.1 to 0.8, and sufficient bone density that was capable of fitting at least four implants, with each implant at least 10 mm in length. This study excluded medically compromised patients and those with severe bone dehiscence and/or fenestrations.

**Figure 1:**
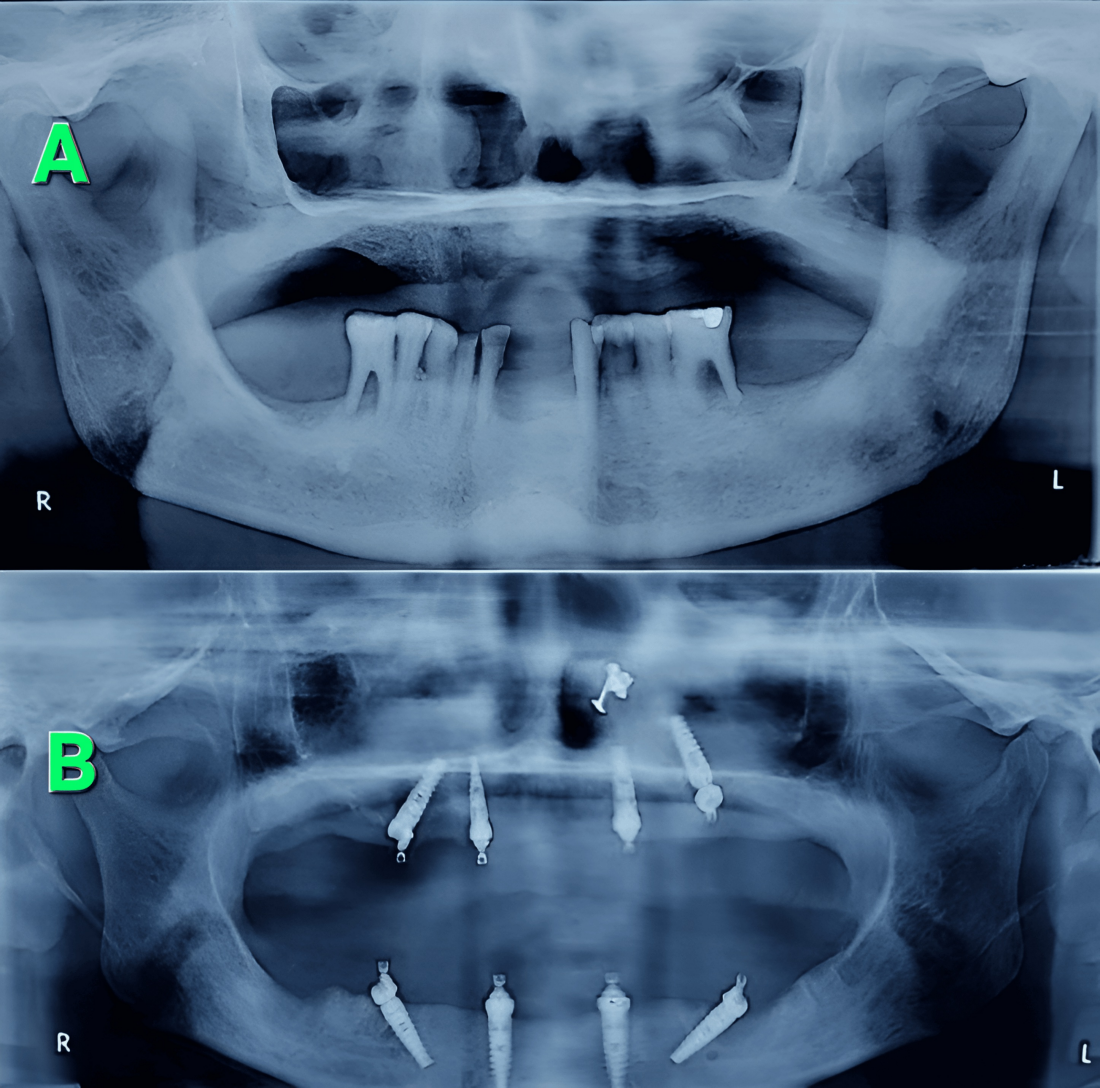
Orthopantomographs before and after the implant procedure A: prior to the start of procedure, B: after insertion of the “all-on-four” implants Source: Original images photo-captured by the first author, Attribution: Dr. Daisy Loyola (first author)

**Figure 2:**
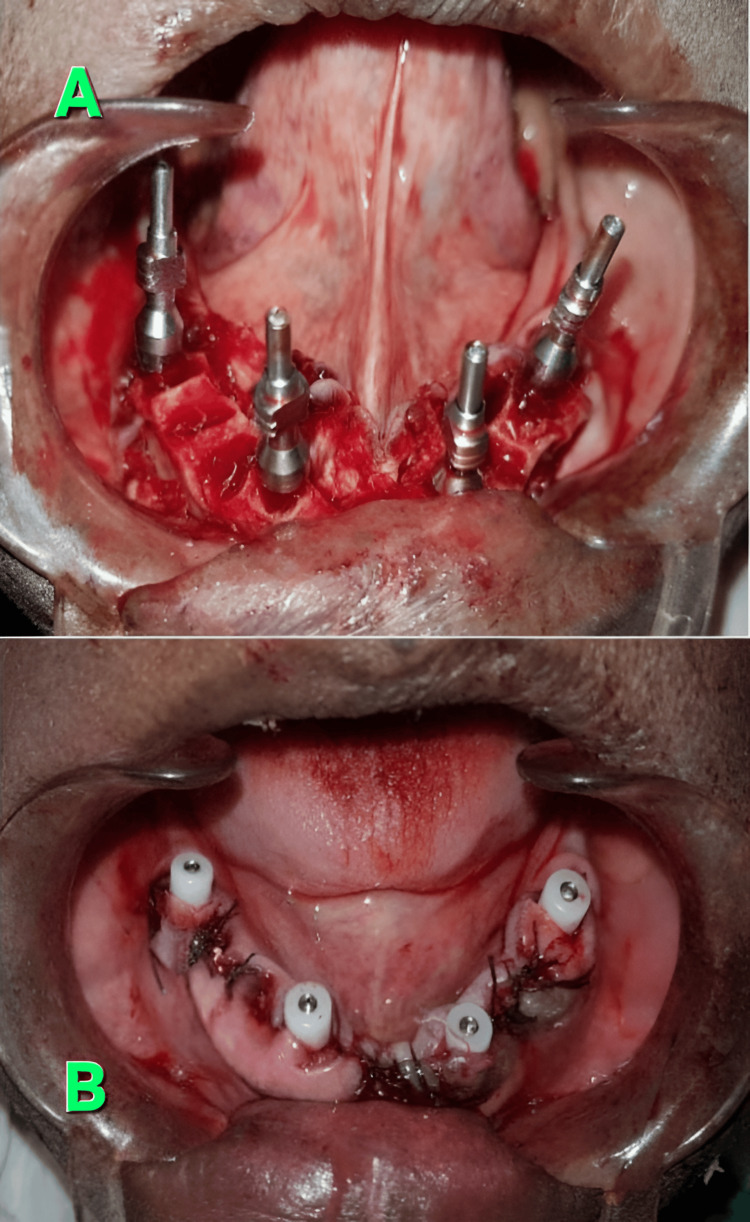
Impression procedure A: four impression copings placed on the implants of the lower arch, B: four healing screws on four implants placed in the mandible once the incision is closed Source: Original images photo-captured by first author, Attribution: Dr. Daisy Loyola (first author)

The surgical procedures were performed under local anesthesia using 2% lignocaine with 1:80,000 adrenaline. Antibiotics were given 1 hour prior to the start of surgery and also for three days during the postoperative period. Prior to the start of the surgical procedures, preoperative orthopantomographs (OPGs) were taken for a general figurative teeth assessment (Figure 1). For all cases, the cone beam computed tomography (CBCT) was also taken for the guidance and proper positioning of the screws during the procedure before placing the implants and also for measurements. Preoperative orthopantomographs (OPGs) helped to preliminarily assess the coping rods and healing screw placements both before and after the impressions (Figures 1, 2). This was mandatorily followed by a postoperative OPG after the placement of implants, also for checking the implants in position (Figure 1). The inter-rater reliability was assessed using Cohen's kappa score. Cohen's kappa score was used to rate the reliability between the observations made by two observers of digital radiographs, and this score was calculated by feeding the digital calibration values to the GraphPad Prism software. A good Cohen's kappa inter-rater reliability score of 0.71 was obtained. In the edentulous arches of patients, the incisions were made on the alveolar crest, from the root of the first molar on one side to that of the root of any convenient molar on the opposite side with bilateral incisions, and then the periosteal reflections were made over the lingual and buccal surfaces of the teeth in their respective arches. In the case of non-edentulous individuals, however, the procedure of incisions was different for the mandibular as well as the maxillary arches. In the mandibular arch region, crestal incisions were placed from the first molar on one side to the first molar on the other side, and a full-thickness mucoperiosteal flap was then raised. Vertical releasing incisions were also given later in order to avoid damage to the mental nerve. In the maxillary arch regions, however, the crestal incisions were placed simultaneously along with two immediate vertical releasing incisions, and then the mucoperiosteal flaps were immediately raised along the buccal aspect of the first molar area region.

This was followed by crestal shaving of the bones in cases of crestal irregularities. Then the “all-on-four implants” were placed and positioned with the help of abutment devices, which included impression rods and healing screws (Figure 2). Four impression rods were placed initially to guide the subsequent four healing screws, and they were positioned at four different quadrants of the arch under consideration (Figure 2). The posteriorly positioned implants were tilted distally at an angle of about 30 degrees to the occlusal plane. The tilted implant placements were initially assisted by a special guide, which was then placed into a 2 mm osteotomy incision that was made at the midline of the jaw, and the titanium guide wire was then contoured into it so that the occlusal central line of the opposing jaw was followed. The preparation for the implants was typically carried out by using a full-length drill depth accompanied by a 2 mm twist drill, followed by widening of the entrance in the cortical bone with subsequent step drills depending upon the bone density and implant diameter. The implant neck was then positioned at the bone interface level, with bicortical anchorage supports whenever possible, and the sockets of the teeth were then debrided to remove all soft tissue remnants. Based upon the degree of irregularity found in the alveolar ridges, alveoloplasty was occasionally done after checking the transitional line between the occlusal plane and the mesial plane. The margins of the flaps were then trimmed to remove the excess tissue, and then sutured later. Postoperative OPGs were taken and compared with the preoperative OPGs to assess the fitment of implants (Figure 1). During the procedure, the values of pocket depths, crestal bone loss, stability quotients, and gingival index scores were noted both before the procedure and three months after the completion of the surgical procedure, and also during the follow-up of the patients at 6, 12, and 18 months, respectively. The inter-observer variability assessment between the observers was done using the ICC (intra-class coefficient score) by using the GraphPad Prism software. A good ICC value of 0.811 was obtained, which highlighted the reliability of observations and results. Before the postoperative assessment of pocket depths and gingival indices, it was ensured that all soft tissue remnants in and around the debrided pockets were thoroughly cleaned and completely removed. For the statistical analysis and interpretation of results, the mean values between two groups were compared using the unpaired t-test, and a p-value of less than 0.05 was considered significant for a type 1 error of 0.8 and for a 95% confidence interval. The p-value interpretation was done using the trial version of the GraphPad Prism software. Only two groups were compared at a time; that is why the unpaired t-test was used to compare two unrelated groups, as the mutual relationships between the two groups were unknown. The tables were plotted using Microsoft Word 2010 (Redmond, USA). The means with standard deviation were calculated using MS Office Excel 2010 version.

## Results

The pocket depths of the teeth that had been fitted with the implants for each of the patients were measured using CBCT (conal beam tomography), and the fitment of the implants within the pockets was assessed using the dental probes as well as OPGs (Figure 1) by three independent observers, and the mean values of the same were noted. The crestal bone thickness of the respective teeth was also measured using digital calipers both before and after the impression procedure (Figure 2) for each of the patients concerned, and the value of the difference in thickness between the readings was then calculated to be the actual crestal bone loss. The mean of the crestal bone loss was noted, tabulated, and compared with the results of the mean pocket depth (Table 1).

**Table 1:** Comparison between mean pocket depth and mean crestal bone loss between maxillary and mandibular implants at different time periods during the “all-on-four” implants procedure mm: Millimeters, SD: Standard deviation, p-value of 0.0053 was measured using an "unpaired t-test". It is significant. (A p-value of less than 0.05 was considered significant)

No. of patients (N)=90	Mean +/- SD of pocket depth (mm)			Mean +/- SD of crestal bone loss (mm)		
Time periods after surgery	Maxillary implants	Mandibular implants	p-value	Maxillary implants	Mandibular implants	p-value
3 months	1.58 +/- 0.02	1.33 +/- 0.05	0.0023	1.63 +/- 0.05	1.35 +/- 0.02	0.0053
6 months	1.67 +/- 0.11	1.38 +/- 0.02		1.74 +/- 0.11	1.40 +/- 0.11	
12 months	1.78 +/- 0.02	1.46 +/- 0.11		1.79 +/- 0.02	1.50 +/- 0.02	
18 months	1.90 +/- 0.05	1.53 +/- 0.02		1.85 +/- 0.11	1.63 +/- 0.11	
24 months	1.80 +/- 0.02	1.53 +/- 0.11		1.80 +/- 0.02	1.63 +/- 0.11	

As evident in Table 1, significant associations were obtained for pocket depths and crestal bone loss, respectively, for each of the maxillary and mandibular arch implants. The results in Table 1 show that the pocket depths are significantly reduced within the roots of the crestal bone when the mandibular implants were placed over their respective arches as compared to the maxillary implants. Also, the crestal bone loss was significantly reduced after the mandibular arch implants were placed over the roots as compared to the maxillary arch implants. The results in Table 1 also revealed that the values of pocket depths of the teeth, as well as the values of crestal bone loss, became stabilized in the mandibular arches after the placement of their respective mandibular arch implants between 18 and 24 months, in contrast to the maxillary arch implants.

The mean of the implant stability quotient values that were obtained using the Osstell device, as observed by three independent observers, was assessed and compared for the maxillary as well as the mandibular arch implants and noted. Similarly, the gingival index scores were also assessed and compared for the upper and lower jaw implants, respectively, and the findings were then tabulated and compared (Table 2).

**Table 2:** Comparison between mean implant stability quotient and mean gingival index between maxillary and mandibular implants at different time periods of the “all-on-4” implants procedure SD: Standard deviation; a p-value of 0.0017 was measured using an "unpaired t-test." It is significant (a p-value of less than 0.05 was considered significant).

No. of patients (N)=90	Mean +/- SD of ISQ (Implant stability quotient)			Mean +/- SD of gingival index		
Time periods after surgery	Maxillary implants	Mandibular implants	p-value	Maxillary implants	Mandibular implants	p-value
3 months	70.23 +/- 0.02	74.20 +/- 0.05	0.0001	2.63 +/- 0.05	0.35 +/- 0.02	0.0017
6 months	71.30 +/- 0.11	74.80 +/- 0.02		1.74 +/- 0.11	0.40 +/- 0.11	
12 months	71.78 +/- 0.02	75.30 +/- 0.11		1.79 +/- 0.02	1.50 +/- 0.02	
18 months	72.40 +/- 0.05	75.83 +/- 0.02		2.85 +/- 0.11	0.63 +/- 0.11	
24 months	71.80 +/- 0.02	75.83 +/- 0.11		1.80 +/- 0.02	0.63 +/- 0.11	

As observed in Table 2, the stability of mandibular implants was significantly higher when compared to the maxillary implants. Also, the gums around the mandibular implants had a lower gingival index as compared to the maxillary implants. The results in Table 2 also revealed that the values of stability quotients of the implanted teeth as well as the values of gingival index in the surrounding gums became stabilized in the mandibular arches after the placement of their respective mandibular arch implants between 18 and 24 months, in contrast to the implants that were placed in the maxillary arch.

## Discussion

As per the research conducted by previous workers such as T. Uesugi et al. and E. L. Agliardi et al., the ‘all-on-four’ technique of dental implants was found to be the most trustworthy technique for a “full-mouth supported prosthesis” with regard to stability for the upper as well as the lower jaws [3,4]. The findings in this study did not allude to the findings of T. Uesugi et al. and E. L. Agliardi et al., as the stability quotients as well as the gingival index scores of both arches after the implant procedures were not uniformly healthy (Table 2). When the researchers of previous studies analyzed the stability of mandibular arch implants over the maxillary implants, they found that the implants that were placed in the maxillary arches gave almost similar results of stability to that of their mandibular counterparts, perhaps because of the lesser density of bone in the maxillae that needed to be supplemented and buttressed by the implants as compared to the mandibles [3,4]. However, the results in this study did not allude to the findings of those of the previous ones as conducted by T. Uesugi et al. and E. L. Agliardi et al., because it was observed in this study that the pocket depths of the mandibular arch implants were definitely less as compared to their maxillary counterparts (Table 1). The variability of the pocket depths in this study was also confirmed during the OPGs as well as during the impressions, followed by crestal shaving of teeth (Figures 1, 2). As per the findings of previous studies done by T. Uesugi et al. and A. Marković et al., the immediate loading of the edentulous maxillae with implants after an extraction was more successful than that with implants fitted after prolonged periods of extraction [5,6]. This was because the degree of resorption of bone was a challenge to the fitment of implants, as also observed by previous workers such as D. Soto-Penaloza et al. [7]. The results of this study, however, partially contradicted the aforementioned findings of previous researchers, because the pocket depths as well as the crestal bone losses for both the arches in this study were not uniform and became evenly stabilized at a static pace only between 18 and 24 months of the procedure, with an initial period of turmoil ranging from 3 to 12 months of the same (Table 1). The maxillary arches, however, had more robust bone losses with fluctuations in their pocket depths as compared to the mandibular arches after a period of 12 months, suggesting a slow but even resorption of bone in the mandibles as compared to the maxillae [6,7]. Also, the pocket depths as well as the crestal bone losses in the mandibular arches were stabilized more profoundly as compared to the maxillary arch implants between 18 and 24 months, further adding strength to the aforementioned discussion in this study (Table 1).

According to the studies done by P. Malo et al., the average marginal bone loss was found to be around 1.5 mm during the first year of functional use of an implant [8]. However, certain other workers, such as M. Taruna et al., had reported the marginal bone loss to range between 0.4 and 1.2 mm for at least one year after the implant surgery [9]. But in this study, the average mean crestal bone loss in the mandible was found to be less than 1.5 mm, and that in the maxilla was slightly more than 1.5 mm (Table 1), which was in stark contrast to the findings of previous researchers [8,9]. As per the studies conducted by other researchers, such as RB Osman et al. and JS Seibert et al., the stability quotients as well as the gingival health of maxillary and mandibular implants were more or less the same after the implant procedure without any comparable significant changes between the two [10,11]. However, the results in this study showed that the mandibular implants had better stability quotients and also better gingival index scores as compared to their maxillary counterparts (Table 2). Perhaps this could be the reason for the decreased pocket depth as well as the decreased crestal bone loss observed in the mandibular implants as compared to the maxillary implants.

As per the study conducted by M. Bevilacqua et al., the gingival indices after implantation in both the upper and lower arches were equally healthy, and they also showed in their study that the placement of implants had no role to play in affecting the oral quality indices of the teeth [12]. The results in this study, however, did not agree with the findings of M. Bevilacqua et al. because it was found in this study that the gingivae around the mandibular arches had a lower and better gingival score as compared to the maxillary arch gingivae after placement of implants (Table 2). This becomes even more important due to the fact that inflamed or unhealthy gingivae may affect the quality of positioning of implants, as shown by G. Sivaramakrishnan et al. in their meta-analysis [13]. This point of the essence of the quality of gingivae after implantation was also highlighted by JA Fernández-Ruiz et al. in their study wherein they had shown that for certain cases the implants of maxillary arches had a better gingival score compared to the mandibular arches, whereas in certain other cases both of them had no changes in their gingival scores [14]. But in this study, however, it was found that the mandibular arches had a better gingival score than the maxillary arch implants, thereby indicating the better adaptability and compatibility of mandibular arch implants to the “all-on-four” technique (Table 2).

Limitations of this study

The follow-up of patients in this study could be done only for a period of 24 months after the completion of the procedure, as the patients were reluctant to further follow-ups due to noncompliance. This could be considered a limitation of this study, as further follow-ups would have yielded better results. Furthermore, the limited sample size in this study could also be considered a limitation, as this study was not conducted in collaboration with centers throughout the country.

## Conclusions

The mean pocket depths as well as the mean crestal bone losses of the all-on-four implants procedure were greater in the maxillary arch implants than in the mandibular arch implants. The implant stability quotient values were higher in mandibular arch implants. Overall, the compatibility of mandibular arches for the “all-on-four implants” technique was better compared to the maxillary arch implants.
